# Dietary Supplementation with Naringin Improves Systemic Metabolic Status and Alleviates Oxidative Stress in Transition Cows via Modulating Adipose Tissue Function: A Lipid Perspective

**DOI:** 10.3390/antiox13060638

**Published:** 2024-05-24

**Authors:** Liuxue Li, Sarula Bai, Huiying Zhao, Jian Tan, Ying Wang, Ao Zhang, Linshu Jiang, Yuchao Zhao

**Affiliations:** 1 Beijing Key Laboratory of Dairy Cow Nutrition, College of Animal Science and Technology, Beijing University of Agriculture, Beijing 102206, China; 202130312014@bua.edu.cn (L.L.); 202130312010@bua.edu.cn (H.Z.); 202230312013@bua.edu.cn (J.T.); 202230321112@bua.edu.cn (Y.W.); 202330321119@bua.edu.cn (A.Z.); 2 Beijing Sunlon Livestock Development Co., Ltd., Beijing 100076, China; saruul_bai@163.com

**Keywords:** adipose tissue, naringin, systemic inflammation, oxidative stress, transition dairy cow

## Abstract

Dairy cows face metabolic challenges around the time of calving, leading to a negative energy balance and various postpartum health issues. Adipose tissue is crucial for cows during this period, as it regulates energy metabolism and supports immune function. Naringin, one of the main flavonoids in citrus fruit and their byproducts, is a potent antioxidant and anti-inflammatory phytoconstituent. The study aimed to evaluate the effects of supplemental naringin on performance, systemic inflammation, oxidative status, and adipose tissue metabolic status. A total of 36 multiparous Holstein cows (from ~21 d prepartum through 35 d postpartum) were provided a basal control (CON) diet or a CON diet containing naringin (NAR) at 30 g/d per cow. Supplemental NAR increased the yield of raw milk and milk protein, without affecting dry matter intake. Cows fed NAR showed significantly lower levels (*p* < 0.05) of serum non-esterified fatty acid (NEFA), C-reactive protein, IL-1β, IL-6, malonaldehyde, lipopolysaccharide (LPS), aspartate aminotransferase, and alanine aminotransferase, but increased (*p* < 0.05) glutathione peroxidase activity relative to those fed CON. Supplemental NAR increased (*p* < 0.05) adipose tissue adiponectin abundance, decreased inflammatory responses, and reduced oxidative stress. Lipidomic analysis showed that cows fed NAR had lower concentrations of ceramide species (*p* < 0.05) in the serum and adipose tissue than did the CON-fed cows. Adipose tissue proteomics showed that proteins related to lipolysis, ceramide biosynthesis, inflammation, and heat stress were downregulated (*p* < 0.05), while those related to glycerophospholipid biosynthesis and the extracellular matrix were upregulated (*p* < 0.05). Feeding NAR to cows may reduce the accumulation of ceramide by lowering serum levels of NEFA and LPS and increasing adiponectin expression, thereby decreasing inflammation and oxidative stress in adipose tissue, ultimately improving their systemic metabolic status. Including NAR in periparturient cows’ diets improves lactational performance, reduces excessive lipolysis in adipose tissue, and decreases systemic and adipose tissue inflammation and oxidative stress. Integrating lipidomic and proteomic data revealed that reduced ceramide and increased glycerophospholipids may alleviate metabolic dysregulations in adipose tissue, which in turn benefits systemic metabolic status.

## 1. Introduction

A successful transition sets the stage for a profitable lactation, with optimal production, reproduction, and health, avoiding premature culling. However, metabolic stress and health issues during this period can significantly impact profitability [1]. Periparturient cows experience metabolic stress similar to human metabolic syndrome, resulting from disrupted nutrient metabolism, leading to dyslipidemia, inflammation, and oxidative stress [2]. Adipose tissue not only stores energy but also acts as an endocrine organ, regulating systemic metabolism and inflammation [3]. Dysregulated lipolysis in adipose tissue is a key factor in periparturient disease risk, triggering inflammation and the infiltration of adipose tissue macrophages [3,4,5]. At the same time, lipolysis enhances free radical production and, when intense and protracted, will predispose the adipose tissue to oxidative stress [6]. By intensifying adipose tissue inflammation, oxidative stress impairs adipocyte response to insulin. Oxidative stress worsens adipose tissue lipolysis and inflammation, creating a harmful cycle where each exacerbates the other [3]. Excessive lipolysis-induced metabolic dysfunction in adipose tissue is thought to contribute to common conditions like fatty liver or ketosis observed in periparturient cows [7].

Many plant-derived polyphenols or flavonoids are known for their anti-oxidative and anti-inflammatory properties, and several studies have evaluated the effects of supplementing transition cows with plant polyphenols or flavonoids. Early-lactation cows receiving green tea and curcuma extract exhibited decreased plasma non-esterified fatty acid (NEFA) relative to that of control cows [8], suggesting that plant polyphenols could inhibit excessive body fat mobilization. Periparturient supplementation of cows with grape seed and grape marc meal extract or green tea and curcuma extract reduced hepatic FGF21 mRNA levels in weeks 1 and 3 of lactation [8,9], indicating reduced liver stress. Grape seed and grape marc meal extract supplementation also lowered plasma levels of serum amyloid A (SAA) and haptoglobin (Hp) [9,10], indicating that plant polyphenols modulated the inflammatory response in dairy cows. Transition cows supplemented with green tea extract showed significant reductions of some genes related to endoplasmic reticulum stress and a trend of lower liver triacylglycerol (TG) concentration [11].

Citrus, a major global crop, is widely used in food processing and fresh juice production. The peel of citrus contains flavanones such as neohesperidin, hesperidin, naringin, and narirutin [12]. As one of the primary constituents of citrus flavonoids, naringin has garnered widespread attention in recent years. Studies have revealed the health-promoting effect of naringin-rich citrus peel extract (naringin > 19%) as a feed supplement in mid-lactation cows [13,14,15,16]. In particular, citrus peel extract supplementation improved ruminal fermentation [16], improved milk yield and milk lactose content, decreased milk somatic cell count (SCC), and improved milk lipid composition and antioxidant capacity in cows [13,14]. In a study by Zhao et al. [15], cows fed a high-starch diet with 100 g/d of citrus peel extract rich in naringin showed an improved hindgut microbiome and host metabolic homeostasis through regulating sphingolipid metabolism.

Additionally, Zhao et al. [17] observed that compared with the control, mid-lactation dairy cows supplemented with citrus peel extract have a greater level of adiponectin, an abundant adipose tissue-derived cytokine. Ying et al. [18] found that the citrus extract lowered the plasma NEFA concentration (6 h after feeding) and enhanced the insulin level (1 h before feeding) in early-lactation and mid-lactation cows, suggesting the extract exhibited some bioactive metabolic properties (i.e., modification of insulin signaling and lipolysis) in adipose tissue. However, whether naringin exerts a health-promoting effect in the adipose tissue of periparturient cows is unknown. The advent of lipidomics technologies has contemporized our understanding of lipid biology and lipotoxicity during the transition period [19]. The identification of changes in glycerophospholipid, sphingolipid, and oxylipid species offers potential biomarkers for detecting abnormal metabolic pathways in transition cows, offering insights for the prevention and treatment of associated disorders [20,21,22].

Therefore, the objectives of the study were to evaluate the effects of naringin on lactational performance and systemic and adipose tissue metabolic status parameters. Integrated lipidomics and proteomics analyses were conducted to reveal the action mechanisms of naringin in adipose tissue. We explored the connection between distinct lipid species in adipose tissue and blood to assess how changes in adipose tissue physiology may influence systemic metabolic shifts induced by naringin supplementation. We hypothesized that supplementing NAR into the diet of transition cows could improve adipose tissue function, decrease inflammatory response and oxidative stress by limiting adipocyte lipolysis, and improve systemic metabolic status and lactational performance.

## 2. Materials and Methods

### 2.1. Experimental Design

All procedures for the use and handling animals were approved by the Institutional Animal Care and Use Committee at Beijing University of Agriculture (Animal Use Protocol # BUA2022052).

In total, 36 multiparous Holstein cows were involved in a randomized complete block design study conducted from August to September 2022 in a commercial farm (Tongzhou District, Beijing, China). Cows were divided by parity, expected calving date, and body condition score (BCS), then randomly assigned within blocks to one of two treatments: (1) a control group receiving the basal total mixed ration (TMR) without addition (CON), or (2) a group receiving basal TMR supplemented with 30 g/d per cow of naringin (NAR, purity ≥ 95%; Xi’an Xuquan Biotechnology Co., Ltd., Xi’an, China). We chose to add 30 g/d per cow of naringin because a previous study we conducted on mid-lactation dairy cows showed that supplementing their diet with 100 g/d of citrus extract (containing approximately 20% naringin) significantly reduced pro-inflammatory cytokine levels and improved lactation performance [17].

Dietary treatments were applied to encompass the close-up dry period (d −21 d before expected parturition) and early-lactation period (d 35 of lactation). Study animals were offered a TMR formulated to meet or exceed the requirements of dry or lactating cows (Table S1) [23].

Diet samples were collected every two weeks to determine DM (ID 934.01), ash (ID 942.05), N (ID 984.13), and ether extract (ID 920.39) content, according to the methods of the Association of Official Analytical Chemists (AOAC) [24]. Neutral detergent fiber was determined following the procedure outlined by Van Soest et al. [25]. Cows were offered TMR twice daily at 0700 and 1500 h ad libitum, prepartum and postpartum. During the treatment period, the additives were incorporated into a fixed quantity of 1 kg of balanced concentrate feed, ensuring the effective administration of the predetermined dose of naringin. The diets were offered at 10% excess to allow ad libitum feed intake.

Dry and fresh cows were kept in separated areas of a freestall equipped with individual feeding gates. Cows with signs of imminent parturition were moved to individual calving pens and, if deemed clinically healthy, proceeded to the lactation group within 24 h of calving. Lactating cows were milked thrice daily at 0500, 1330, and 2000 h in an eight single milking parlor. All cows had free access to drinking water. Clinical diseases were diagnosed and recorded by a veterinarian or experienced farm staff.

### 2.2. Sample Collection

Individual milk yield was measured for each cow at every milking using a milk metering system (Alpro, DeLaval, Tumba, Sweden). Weekly milk samples were collected to analyze milk composition, including fat, protein, lactose, milk urea nitrogen (MUN), and SCC, using a Milkoscan FT 6000 device (Foss Electric, Hillerød, Denmark). Blood was obtained after morning feeding between 08:00 and 10:00 by puncture of a jugular vein, and collected in tubes containing pro-coagulant (BD Vacutainer, Preanalytical Solutions, Franklin Lakes, NJ, USA) on d − 7 before the expected calving date and on d 2, 14, and 28 postpartum. Blood was centrifuged at 2000× *g* at room temperature for 15 min within 20 min of collection, and serum was kept at −20 °C until analysis. Subcutaneous adipose tissue samples were collected from the tail-head area, alternating between the right and left sides, at 2, 14, and 28 days after calving. Due to ethical considerations, biopsies were limited to 16 cows, with 8 cows from each of the CON and NAR groups. Before the procedure, the biopsy sites were prepared by clipping the hair and sterilizing the skin with iodine soap and water. Following aseptic preparation with iodine scrub and 70% alcohol, local anesthesia was administered using a 2% lidocaine solution. After a 10 min wait, a 2 cm skin incision was made, and approximately 3 g tissue samples were collected. The samples were rinsed with sterile saline to remove excess blood and immediately stored in screw-capped microcentrifuge tubes. They were then snap-frozen in liquid nitrogen and preserved at −80 °C for further analysis. The health of the cows was monitored for 7 days post-surgery, with surgical clips removed after 7 days. No antibiotics were administered following the biopsy procedure.

### 2.3. Blood Metabolic Status Parameters

Serum NEFA (enzymatic colorimetric assay, #AC10172) and β-hydroxybutyric acid (BHBA; enzymatic colorimetric assay, #AC10788) were determined with the commercial kits (Shanghai Acmec Biochemical Technology Co., Ltd., Shanghai, China).Serum glucose (glucose oxidase method, #A154-1-1), insulin (bovine-specific ELISA kit, #H203-1-1), TG (GPO-PAP method, #A110-1-1), total cholesterol (TC; GPO-PAP method, #A111-1-1), low-density lipoprotein cholesterol (LDL-C; enzymatic colorimetric assay, #A113-1-1), leptin (bovine-specific ELISA kit, #H174-1-2), total protein (TP; coomassie brilliant blue method, #A045-2-2), albumin (ALB; bromocresol green method, #A028-2-1), lipopolysaccharide (LPS; bovine-specific ELISA kit, #H255-1-1), C-reactive protein (CRP; immunoturbidimetry assay, #E023-1-1), IL-1β (bovine-specific ELISA kit, #H002-1-2), IL-2 (bovine-specific ELISA kit, #H003-1-2), IL-6 (bovine-specific ELISA kit, #H007-1-2), IL-10 (bovine-specific ELISA kit, #H009-1-2), and TNF-α (bovine-specific ELISA kit, #H052-1-2) were determined using commercially available kits (Nanjing Jiancheng Bioengineering Institute, Nanjing, China). Serum adiponectin (bovine-specific ELISA kit, #SEKB-0196), aspartate aminotransferase (AST; colorimetric assay, #BC1565), alanine aminotransferase (ALT; colorimetric assay, #BC1555), superoxide dismutase (SOD; WST-1 method, #BC5165), glutathione peroxidase (GSH-Px; colorimetric assay, #BC1195), and malonaldehyde (MDA; colorimetric assay, #BC0025) were analyzed with commercially available kits (Beijing Solarbio Science & Technology Co., Ltd., Beijing, China). The biochemical parameters were assessed following the manufacturer’s guidelines. Absorbance readings were obtained using a microplate reader (Multiskan FC; Thermo Fisher, New York, NY, USA). Coefficients of variation within and between assays were lower than 10%, in all cases.

### 2.4. Biochemical Parameters Analysis of Subcutaneous Adipose Tissue

Adipose tissue protein was extracted using RIPA buffer (Thermo) supplemented with protease inhibitor cocktail (Roche, Indianapolis, IN, USA) and quantified through the BCA Assay (Thermo). Pro-inflammatory factors (IL-1β, IL-6, TNF-α), oxidative markers (total antioxidant capacity (T-AOC), SOD), NLRP3 inflammasome (NLRP3, ASC), and adiponectin levels were determined via commercial and bovine-specific ELISA kits (Nanjing Jiancheng Bioengineering Institute, Nanjing, China).

### 2.5. Serum and Adipose Tissue Lipiomics Analysis

Serum and adipose tissue samples obtained on d 14 from 16 cows were analyzed for lipidome. Serum total lipids were extracted using a previously described method [26]. Briefly, after centrifugation (2100× *g*, 5 min), 50 μL of supernatant was homogenized in 1 mL of methyl-tert-butyl-ether (MTBE): methanol mix (3:1, *V*/*V*). After adding 200 μL of water, the mixture was vortexed for 1 min before being centrifuged at 12,000 rpm for 10 min. After centrifugation, 500 μL of the supernatant was then transferred to a 2 mL centrifuge tube and concentrated using nitrogen blowing. Adipose tissue lipids were extracted following the protocol of Lam et al. [27]. In brief, adipose tissues (40 ± 5 mg) were homogenized in 200 μL deionized water. The homogenate was mixed with 240 μL of ice-cold methanol for 1 min. Next, 800 μL of MTBE was added, and the homogenate was vortexed and equilibrated at room temperature for 20 min. Afterwards, the mixture was centrifuged at 8000× *g* for 15 min at 10 °C. The upper layer was collected and transferred to another 1.5 mL tube and evaporated to dryness under a gentle stream of nitrogen. The lipid extracts of the serum and adipose tissue samples were dissolved in a mixture of acetonitrile, isopropanol, chloroform, and water (35:35:20:10 *v*/*v*/*v*/*v*) and analyzed using Orbitrap high-resolution tandem mass spectrometry (Q Exactive HF, Thermo Scientific, Waltham, MA, USA) coupled with a Vanquish ultra-high performance liquid chromatography (UHPLC) system (Thermo Scientific). Separation of lipids was achieved on an ACQUITY UPLC-HSS C18 column (2.1 mm × 150 mm × 2.5 μm; Waters, Milford, MA, USA) using a mobile phase consisting of acetonitrile/water (60:40, *v*/*v*, 0.1% formic acid, 10 mmol/L ammonium formate) and isopropanol/acetonitrile (90:10, *v*/*v*, 0.1% formic acid, 10 mmol/L ammonium formate). The mobile phase flow rate was 0.4 mL/min, with a gradient of solvent B (acetonitrile/isopropanol) starting at 40% and reaching 99% at 18 min, followed by re-equilibration. The sample injection volume was 2 μL.

Lipid annotation involved matching the retention time (RT), accurate mass, and fragmentation ion pattern against an in-house lipidomics database, along with major public spectral libraries, like the LIPID MAPS structure database (LMSD) and the Human Metabolome Database (HMDB). Orthogonal partial least squares discriminant analysis (OPLS-DA) was used to visualize metabolome differences among treatments. Variable importance in projection (VIP) values from OPLS-DA determined differentially abundant lipid species. The criteria for selecting significantly different lipids included VIP > 1, *p* < 0.05, and FC > 1. The differentially identified metabolites and lipids were uploaded to Metaboanalyst 5.0 (http://www.metaboanalyst.ca/; accessed on 12 December 2023), along with the KEGG database, for pathway analysis. The correction of *p* values in the metabolome and lipidome was performed using the false discovery rate (FDR) error control method. Statistical significance was set at *p* < 0.05.

### 2.6. Adipose Tissue Proteomics Analysis

Adipose tissue samples obtained on d 14 from 16 cows was analyzed for proteome. Protein extraction from adipose tissue samples followed the method of Zhang et al. [28], with minor modifications. Briefly, adipose tissue (60 mg) was lysed in 300 μL of lysis buffer (7 M urea, 2 M thiourea, 0.1% CHAPS, and a proteasome inhibitor). The tissue was then fully ground using abrasive beads (70 HZ, 2 min) and centrifuged at 12,000× *g* for 30 min at 4 °C to collect the supernatant. The protein concentration was analyzed using a Pierce™ BCA assay (Thermo Scientific, Waltham, MA, USA). Protein digestion followed the filter-aided sample preparation method [29]. After reduction with 20 mM dl-dithiothreitol and alkylation with 50 mM iodoacetamide, samples were digested with 2% trypsin at 37 °C for 12 h. Peptide samples were then collected for MS analysis and resuspended in 0.2% trifluoroacetic acid and 2% acetonitrile in water before LC–MS/MS analysis.

Peptide digests from each sample underwent analysis on the timsTOF Pro, a trapped ion mobility Q-TOF mass spectrometer. This analysis included online separation utilizing a C18 column (nanoACQUITY column Peptide BEH C18, 75 μm × 250 mm) coupled with a nanoElute nano-flow UHPLC system (Bruker Daltonics, Billerica, MA, USA) operating at 250 nL/min. Separation was achieved using a 120 min organic gradient ranging from 2% to 85% mobile phase B (mobile phase A: 0.1% formic acid; mobile phase B: 0.1% FA in acetonitrile). MS spectra were collected within the 400 to 1600 *m*/*z* range at a resolution of 120,000, with a maximum injection time of 100 ms and a dynamic exclusion of 15 s. Fragmentation of the top 20 precursors was accomplished using higher-energy C-trap dissociation (HCD) at a normalized collision energy of 27%.

The MS/MS raw data files underwent processing using MaxQuant (version 1.6.2.3) with the integrated Andromeda search engine, which searched against a UniProt protein database of Bos taurus. The search parameters included: (1) allowance of up to two missed cleavage sites for enzyme digestion; (2) a peptide length requirement of more than six amino acids; (3) the alkylation of cysteine (C) as a fixed modification; (4) variable modifications, including oxidation, acetylation, and deamination of methionine (M); (5) a requirement of an FDR of <0.01 for peptides and proteins; and (6) the utilization of razor and unique peptides for protein quantification.

Proteins with blank values in more than 20% of all samples were excluded. Remaining blank values were interpolated using the k-nearest neighbors algorithm implemented in the impute function of R software (version 4.1.2). The complete protein expression profile was then log normalized. Differentially expressed proteins (DEP) were identified using a Wilcoxon rank-sum test with a significance threshold of *p* < 0.05 and FC > 1.5. KEGG, and Gene Ontology (GO) tools were utilized to annotate the biological roles and enriched molecular pathways of DEP. Protein–protein interaction (PPI) networks were constructed using STRING 11.5 to explore connections among DEP.

### 2.7. Calculation and Statistical Analysis

Energy-corrected milk (ECM) and fat-corrected milk (FCM) were determined following the methods described by Tyrrell and Reid [30] and VanBaale et al. [31]. The equations used for calculation were as follows: ECM = (0.327 × milk yield) + (12.95 × fat yield) + (7.2 × protein yield) and 3.5% FCM = (0.432 × milk yield) + (16.23 × fat yield). Feed efficiency was assessed by calculating milk yield divided by dry matter intake (DMI), ECM divided by DMI, and FCM divided by DMI.

The data concerning the biochemical parameters of serum and adipose tissue, as well as lactation performance, were subjected to analysis using the MIXED procedure of SAS (SAS Institute, Inc., Cary, NC, USA). Milk yield and composition data were averaged weekly before statistical analysis. Fixed effects included dietary treatments, time, and the interaction between treatment and time. Block and residual error were treated as random effects. Non-normally distributed data were transformed using the Box–Cox transformation method, where the appropriate lambda value was determined through a Box–Cox transformation analysis conducted with the TRANSREG procedure of SAS.

Data with repeated measures, including serum and adipose tissue biochemical parameters and production data, were subjected to repeated measures analysis. Equally spaced variables were analyzed with various covariate structures: unstructured (UN), variance components (simple), compound symmetry (CS), heterogeneous CS, first-order autoregressive (AR(1)), and heterogeneous (ARH(1)). The optimal covariance matrix was selected based on the smallest Bayesian information criterion (BIC) value. For repeated measures with unequal sampling time intervals, the spatial power covariance structure was utilized. Responses with a single measurement per cow were analyzed, with models incorporating the fixed effect of treatment and the random effect of blocking. Degrees of freedom were adjusted using the Kenward–Roger method. Mean differences were deemed significant at *p* ≤ 0.05 and considered tendencies at 0.05 < *p* ≤ 0.10.

## 3. Results

### 3.1. Feed Intake, Milk Production and Composition

Prepartum DMI reduced as calving approached (Time, *p* < 0.001; Table 1), and DMI gradually increased from d 1 to 35 after parturition for all cows (*p* < 0.01). However, neither prepartum nor postpartum DMI differed between treatments (*p* > 0.50), and no interaction effect between dietary treatments and time was observed. Cows fed NAR had a greater yield of milk (*p* = 0.038) and protein (*p* = 0.002) than those fed with CON. No treatment differences were detected for ECM, FCM, milk fat, or MUN (*p* > 0.10). Feeding NAR tended to increase the content of protein (*p* = 0.086) and lactose (*p* = 0.061) in the milk. Compared with CON cows, cows fed NAR exhibited lower milk SCC (*p* = 0.018). Regarding the feed efficiency, a tendency for a higher (*p* = 0.079) ratio of ECM/DMI was observed in cows fed NAR, but cows in this group exhibited a greater (*p* = 0.028) milk yield/DMI ratio compared with that of CON cows.

### 3.2. Serum Metabolic Status Parameters

The main effects and interactions for serum glucose and lipid metabolism biomarkers are presented in Table 2. There were time effects (*p* < 0.05) for NEFA, BHBA, glucose, insulin, TG, TC, LDL-C, and adiponectin; however, no treatment and time interactions were detected in these indices (*p* > 0.18). Cows fed NAR had lower serum NEFA concentrations than those fed with CON (*p* = 0.028; Table 2). Treatments did not affect the serum concentration of BHBA, glucose, insulin, TG, TC, LDL-C, leptin, or adiponectin (*p* > 0.17).

The main effects and interactions for liver function indices are presented in Table 3. Feeding with NAR tended to decrease serum TP content (*p* = 0.056). The cows fed with NAR exhibited a lower AST (*p* = 0.012) and ALT (*p* = 0.002) activity compared to those fed with CON. Serum TP, AST, and ALT were influenced by time (*p* < 0.02) and the interaction of treatment and time (*p* < 0.02). Cows fed with NAR compared to those fed with CON exhibited a lower serum TP (*p* = 0.012) on d −7 relative to calving, while no significant differences between the two groups were observed after calving. Cows fed with NAR relative to CON had lower serum ALT on d 2 and d 14 postpartum, while no significant differences between the two groups were observed on d −7 (*p* < 0.001) and d 28 (*p* = 0.015) relative to calving. Cows fed with NAR had lower serum AST on d 14 (*p* = 0.036) and d 28 (*p* = 0.001) after calving, but numerically higher AST on d −7 (*p* = 0.438) compared with those fed with CON.

The main effects and interactions for serum endotoxin, antioxidant status, and proinflammatory factors are presented in Table 4. All parameters were affected by time (*p* < 0.02), except for IL-1β, IL-2, and TNF-α. Serum LPS was lower (*p* < 0.001) in cows fed NAR than with in CON cows. We detected a Trt × Time interaction (*p* < 0.001) on serum LPS. On d −7 relative to calving, feeding NAR compared with CON decreased concentrations of serum LPS (*p* < 0.001), whereas the values were similar from calving to d 28 postpartum (*p* > 0.05). Dietary supplementation with NAR decreased serum MDA concentration (*p* = 0.010) and increased the activity of GSH-Px (*p* = 0.029). A trend (*p* = 0.062) was observed for a greater activity of SOD in cows fed with NAR compared to those fed with CON. Feeding the NAR diet decreased serum CRP (*p* = 0.019), IL-1β (*p* = 0.004), and IL-6 (*p* < 0.001) compared with the CON diet. Cows fed the NAR had a greater serum IL-10 concentration (*p* = 0.012) than those fed with CON. There was no difference (*p* > 0.10) in serum IL-2 and TNF-α between the CON and NAR groups.

### 3.3. Adipose Tissue Biochemical Parameters

The main effects and interactions of the adipose tissue biochemical parameters are presented in Table 5. All parameters were affected by time (*p* ≤ 0.003). Cows fed NAR had lower concentrations of IL-1β (*p* < 0.001) and IL-6 (*p* = 0.012) compared to those fed with CON. Dietary treatment did not affect the adipose tissue content of TNF-α (*p* = 0.138). The cows fed with NAR showed a greater T-AOC (*p* < 0.001) activity and tended to have a higher level of SOD (*p* = 0.064) in adipose tissue compared with that of the CON cows. An interaction between treatment and time was detected for T-AOC in the postpartum period (*p* = 0.037), and cows fed with NAR had a greater T-AOC on d 14 after calving (*p* = 0.028). Feeding NAR to cows decreased the content of ASC (*p* = 0.002) and NLRP3 (*p* = 0.050) in adipose tissue relative to feeding them with CON.

### 3.4. Serum and Adipose Tissue Lipidome

Lipid class composition data for serum and adipose tissue samples are presented in Figure S1a and S1b, respectively. The LC–MS analysis allowed for the identification of 582 lipid species, divided into 31 lipid classes, from the serum lipidome, belonging mainly to the glycerolipid (GL), sphingolipid (SP), and glycerophospholipid (GP) groups. Phosphatidylcholine (PC) was the most abundant species, followed by methylphosphatidylcholine (MePC), TG, and sphingomyelin (SM) (Figure S1a). We detected 832 lipid species in the adipose tissue samples (Figure S1a). TG was the most abundant lipid class, followed by PC, phosphatidylethanolamine (PE), and ceramide (Cer).

**Figure 1:**
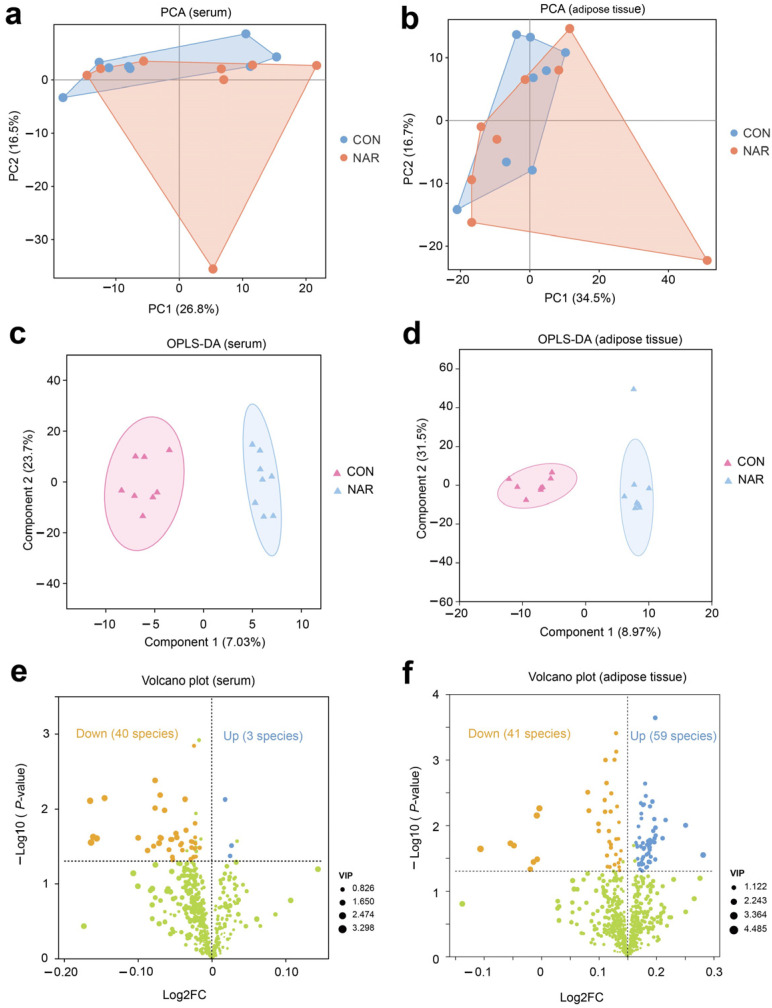
Serum and adipose tissue lipidomes. Principal component analysis (PCA) of (**a**) serum and (**b**) adipose tissue lipidomics. Orthogonal partial least squares discriminant analysis (OPLS-DA) of (**c**) serum and (**d**) adipose tissue lipidomics. Volcano plot of identified lipid species in (**e**) serum and (**f**) adipose tissue. Yellow symbols: significantly decreased lipid species (variable importance in projection (VIP) > 1, *p* < 0.05). Blue symbols: significantly increased lipid species (VIP > 1, *p* < 0.05). CON, control; NAR; naringin; FC, fold change.

Initially, we performed a comparative analysis of the lipidomics data utilizing PCA. The PCA plots indicate that CON and NAR samples do not exhibit distinct separation for serum and adipose tissue samples (Figure 1a,b). We further used OPLS-DA models to determine the differences between CON and NAR. Multivariate OPLS-DA analysis demonstrated distinct differentiation between the lipidomes of CON and NAR (Figure 1c,d). To explore the alterations in serum and adipose tissue lipidomes resulting from dietary NAR, we generated volcano plots using the lipidomic data. Lipids with significant changes were identified based on *p*-values ≤ 0.05 and a VIP > 1 threshold.

There were three lipid species (i.e., Hex2Cer(d16:0/18:1), PS(20:0/18:1), MGDG(18:0e/15:0)) that were significantly upregulated and 40 species (e.g., MePC(8:1e/21:0), PC(12:0e/20:4), PE(16:0p/22:5), DG(20:0/16:0)) that were significantly downregulated in the serum of cows fed with NAR compared to those fed with CON (Figure 1e and Table S2). The volcano plot illustrates that 59 lipids increased (e.g., TG(15:0/6:0/16:1), PS(19:0/18:1), PC(18:0/17:0), PS(22:0/20:3)) and 41 lipids decreased (e.g., TG(16:0/14:0/17:1), TG(16:1/6:0/14:1), Hex1Cer(d18:1/18:0), Hex1Cer(d18:0/18:1)) in cows fed with NAR relative to CON cows (Figure 1f and Table S3).

**Figure 2:**
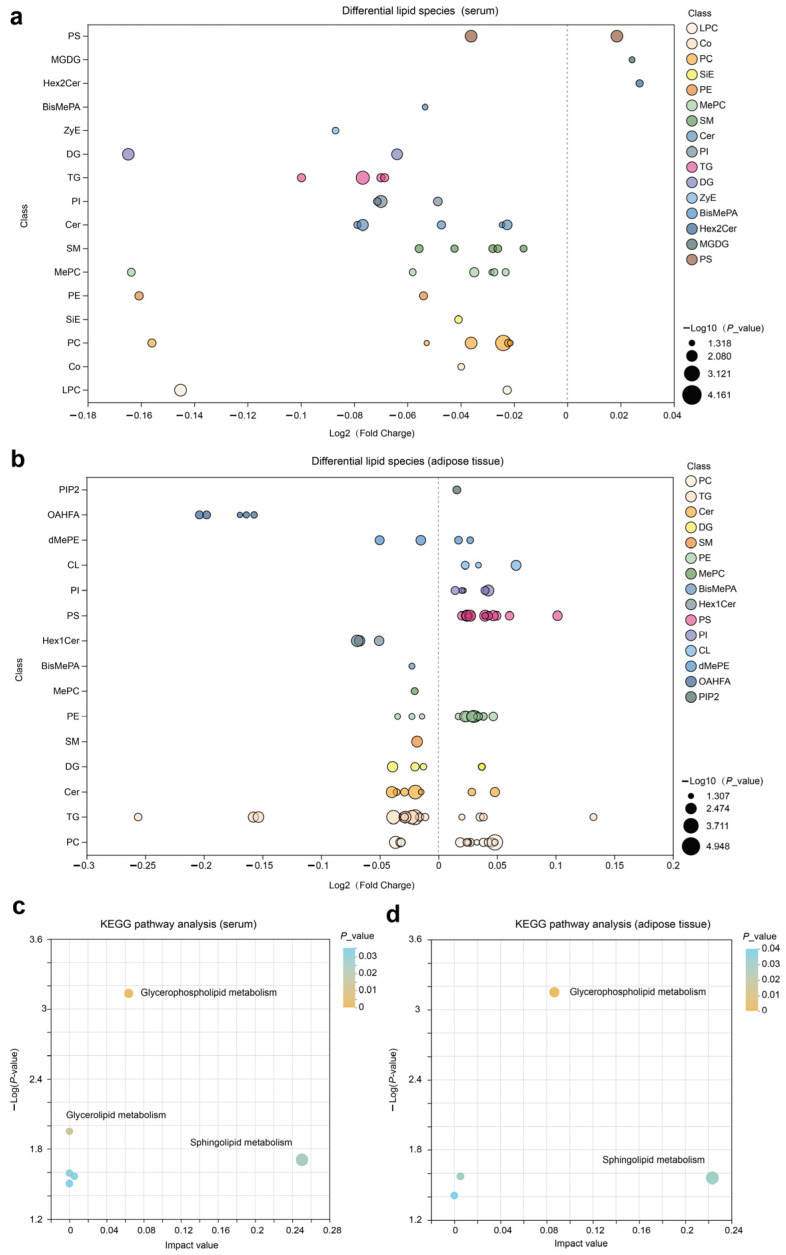
Lipidomic alteration in serum and adipose tissue samples. Differential lipid species in (**a**) serum and (**b**) adipose tissue are presented in the fold-change of NAR relative to CON. KEGG pathway analysis of differential lipid species in (**c**) serum and (**d**) adipose tissue samples. BisMePA, bismethyl phosphatidic acid; Cer, ceramide; CL, cardiolipin; Co, coenzyme; DG, diglyceride; Hex2Cer, dihexosylceramide; LPC, lysophosphatidylcholine; MePC, methylphosphatidylcholine; MGDG, monogalactosyldiacylglycerol; OHAFA, (O-acyl)-1-hydroxy fatty acid; PC, phosphatidylcholine; PE, phosphatidylethanolamine; PI, phosphatidylinositol; PIP2, phosphatidylinositol 4,5-bisphosphate; PS, phosphatidylserine; SiE, sitosteryl ester; SM, sphingomyelin; TG, triglyceride; ZyE, zymosteryl.

We constructed a bubble chart to visually display the differences between CON and NAR regarding various classes of lipids. A decrease in Cer, SM, MePC, PC, TG, PI, and PE in serum samples was observed with cows fed NAR (Figure S2a and Figure 2a). By contrast, we found a significantly increased level of glycerophospholipids (CL, PC, PE, PI, and PS) in adipose tissue of NAR compared with CON cows (Figure S2b and Figure 2b). We also observed significantly decreased glycerolipid (DG and TG) levels in cows fed with NAR compared with CON cows. Among the lipids showing significant statistical differences in adipose tissue, sphingolipids (Cer, Hex1Cer, and SM) drew our attention. Similar to the changes observed in SP in serum, the levels of certain SP species in the adipose tissue of cows fed with NAR also showed a significant decrease compared to those fed with CON. KEGG pathway analysis indicated significant enrichment of glycerophospholipid metabolism and sphingolipid metabolism pathways in both serum (Figure 2c) and adipose tissue (Figure 2d).

**Figure 3:**
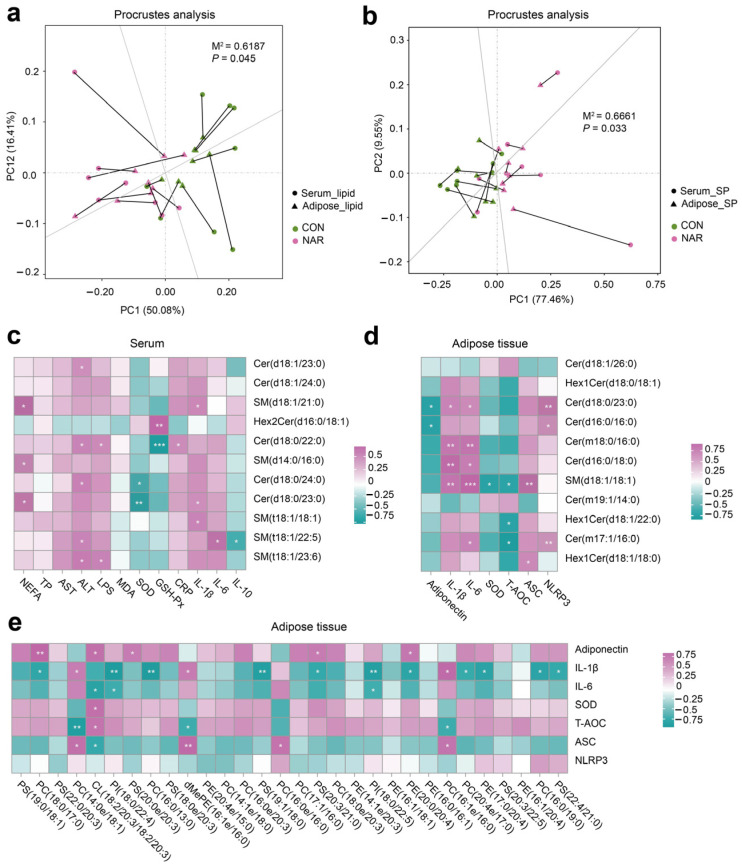
Association analysis of the serum and adipose tissue lipidome. (**a**) The correlation between differential lipid species in the serum and adipose tissue samples by Procrustes analysis; (**b**) the correlation between differential sphingolipid (SP) species in serum and adipose tissue samples by Procrustes analysis; (**c**) Pearson’s correlation between serum differential sphingolipids and serum biochemical parameters; (**d**) Pearson’s correlation between adipose tissue differential sphingolipids and adipose tissue biochemical parameters; (**e**) Pearson’s correlation between adipose tissue differential glycerophospholipids (top 30 of VIP value) and adipose tissue biochemical parameters. ALT, alanine aminotransferase; ASC, apoptosis-associated speck like protein containing a CARD; AST, aspartate aminotransferase; Cer, ceramide; CL, cardiolipin; CON, control; CRP, C-reactive protein; dMePE, dimethylphosphatidylethanolamine; GSH-Px, glutathione peroxidase; Hex2Cer, dihexosylceramide; IL, interleukin; LPS, lipopolysaccharide; MDA, malonaldehyde; NAR, naringin; NEFA, non-esterified fatty acid; NLRP3, NOD-like receptor thermal protein domain-associated protein 3; PC, phosphatidylcholine; PE, phosphatidylethanolamine; PI, phosphatidylinositol; PS, phosphatidylserine; SM, sphingomyelin; SOD, superoxide dismutase; SP, sphingolipid; TP, total protein.

We conducted a correlation analysis to assess the relationship between differential lipids and phenotypes. The Procrustes analysis within all samples from different groups was used to assess the overall associations between the serum lipidome and adipose tissue lipidome. The correlation is considered significant when *p* < 0.05. There was a consistent and significant inter-omics relationship between serum differential lipids and adipose tissue differential lipids across the different groups (Figure 3a; M2 = 0.62, *p* = 0.045), indicating that the lipid metabolism in the adipose tissue metabolic might alter the lipidomic profile of the serum. Serum differential SP showed a significant correlation with differential SP in adipose tissue (Figure 3b; M2 = 0.67, *p* = 0.033). The heatmap of the correlation between serum differential SP and the serum biochemical parameters is shown in Figure 3c. Statistical significance (*p* < 0.05) and the Pearson correlation coefficient (|r|> 0.5) indicate the correlations. Serum NEFA was positively correlated with SM(d18:1/21:0), SM(d14:0/d16:0), and Cer(d18:0/23:0). Serum ALT was positively correlated with Cer(d18:1/23:0), Cer(d18:0/22:0), Cer(d18:0/24:0), SM(t18:1/22:5), and SM(t18:1/23:6). Serum IL-1β was positively correlated with SM(d18:1/21:0), Cer(d18:0/23:0), and SM(t18:1/18:1). Serum SOD was negatively correlated with Cer(d18:0/24:0) and Cer(d18:0/23:0). The heatmap of the correlation between the adipose tissue differential SP and the adipose tissue biochemical parameters was shown in Figure 3d. The adipose tissue content of adiponectin was positively correlated with Cer(d18:0/23:0) and Cer(d16:0/16:0). The adipose tissue levels of IL-1β and IL-6 were positively correlated with Cer(d18:0/23:0), Cer(m18:0/16:0), Cer(d16:0/18:0), and SM(d18:1/18:1). The level of T-AOC was negatively correlated with SM(d18:1/18:1), Hex1Cer(d18:1/22:0), and Cer(m17:1/16:0). We also observed that some SP species were positively correlated with ASC and NLRP3 levels in adipose tissue (Figure 3e). In addition, adiponectin and ASC were positively correlated with some GP species. The levels of IL-1β, IL-6, and T-AOC were negatively correlated with some GP species.

### 3.5. Adipose Tissue Proteome

**Figure 4:**
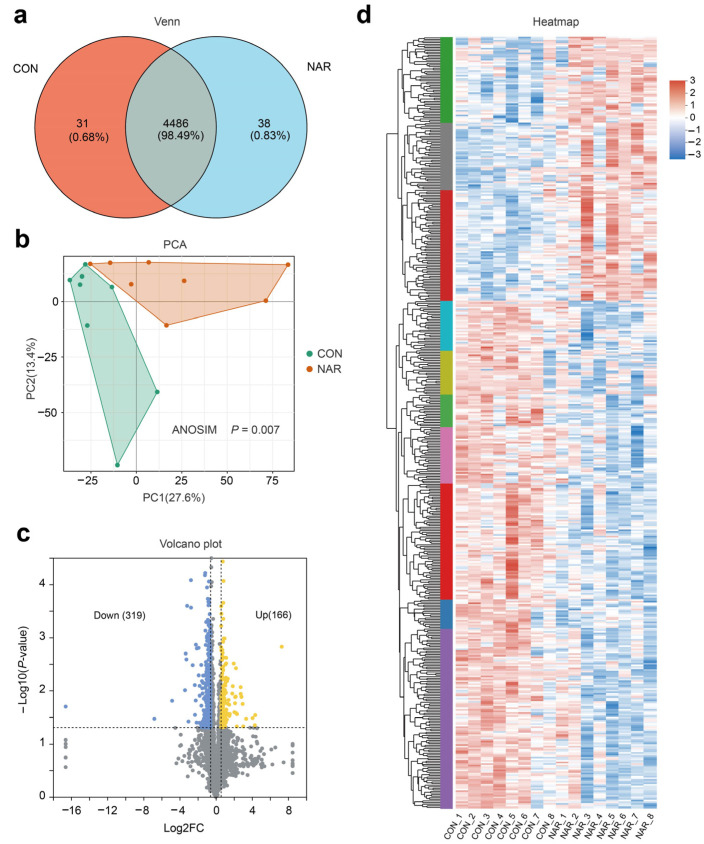
Adipose tissue proteome analysis. (**a**) Venn diagram of the identified proteins; (**b**) PCA score plots of proteome data; (**c**) volcano plot of the proteome; (**d**) heatmap of differentially expressed proteins. CON, control; NAR, naringin; FC, fold change.

After removing the low-scoring spectra, we obtained a total of 25,843 unique peptides and 4546 proteins in the CON and NAR groups through the DIA analysis. Accounting for 98.49% of identified proteins, 4486 were recognized as common proteins from both the CON and NAR groups (Figure 4a). As shown in the PCA plots, the proteins of both the CON and NAR groups were distinctly clustered in the comparisons (Figure 4b). Statistical analysis of similarity (ANOSIM) between groups demonstrated a significant difference (*p* = 0.007) between the proteomics profile of the adipose tissue of the CON and NAR groups. A total of 166 proteins were significantly upregulated, and 319 proteins were significantly downregulated in cows fed with NAR compared with CON cows (Figure 4c). The significantly altered proteins are shown in Figure 4d and Table S4.

**Figure 5:**
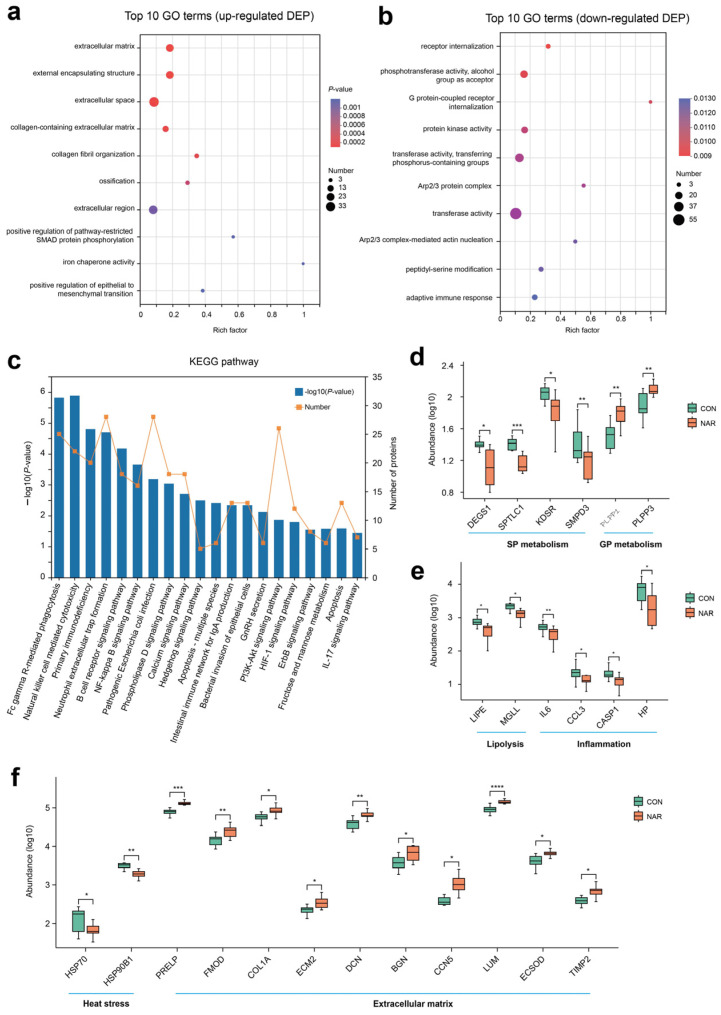
Proteomic alteration in adipose tissue samples. GO enrichment analysis of (**a**) upregulated differentially expressed proteins (DEP) and (**b**) downregulated DEP; (**c**) KEGG enrichment analysis of DEP; (**d**) selected DEP related to sphingolipid metabolism and glycerophospholipid metabolism; (**e**) selected DEP related to lipolysis and inflammation; (**f**) selected DEP related to heat stress and extracellular matrix. * *p* < 0.05, ** *p* < 0.01, *** *p* < 0.001, **** *p* < 0.0001. CON, control; NAR, naringin.

The analysis of the GO functional enrichment of DEP was used to identify the GO functional items. For upregulated DEG, multiple GO terms, such as extracellular matrix (GO:0031012), external encapsulating structure (GO:0030312), extracellular space (GO:0005615), collagen-containing extracellular matrix (GO:0062023), and collagen fibril organization (GO:0030199), were significantly enriched (Figure 5a). Decreases in receptor internalization (GO:0031623), phosphotransferase activity, alcohol group as acceptor (GO:0016773), G protein-coupled receptor internalization (GO:0002031), protein kinase activity (GO:0004672), transferase activity, and transferring phosphorus-containing groups (GO:0016772) were among the top 10 terms prioritized by GO analysis (Figure 5b). Through KEGG enrichment analysis, 485 DEPs were found to be annotated to 304 different metabolic pathways. Pathways related to the immune response and inflammation, such as natural killer cell mediated cytotoxicity, primary immunodeficiency, neutrophil extracellular trap formation, B cell receptor signaling pathway, and the NF-kappa B signaling pathway, were also enriched (Figure 5c).

Based on the phenotypes and lipid profiles of cow serum and adipose tissue, DEP associated with sphingolipid metabolism, glycerophospholipid metabolism, lipolysis, and inflammation are shown in Figure 5d,e. Compared with CON, cows fed with NAR showed a lower abundance of DEGS1, SPTLC1, KDSR, and SMPD3 in adipose tissue (Figure 5d). The abundance of PLPP1 and PLPP3 was greater in cows fed with NAR than in CON cows. Two proteins related to lipolysis (i.e., LIPE and MGLL) and four proteins (i.e., IL6, CCL3, CASP1, and HP) related to inflammation were upregulated in cows fed with NAR compared to those fed with CON (Figure 5e). We also observed that two heat stress-related proteins (i.e., HSP70 and HSP90B1) were downregulated (Figure 5f). As a consequence of the substantial decrease in adipose tissue mass within the initial three weeks postpartum (exceeding 20%, as reported by Akter et al. [32]), significant alterations occur in the gene networks regulating the extracellular matrix (ECM) structure of adipose tissue. We observed that some proteins related to ECM were upregulated in cows fed with NAR compared to those fed with CON (Figure 5f).

### 3.6. Integrated Pathway and Network Analysis

**Figure 6:**
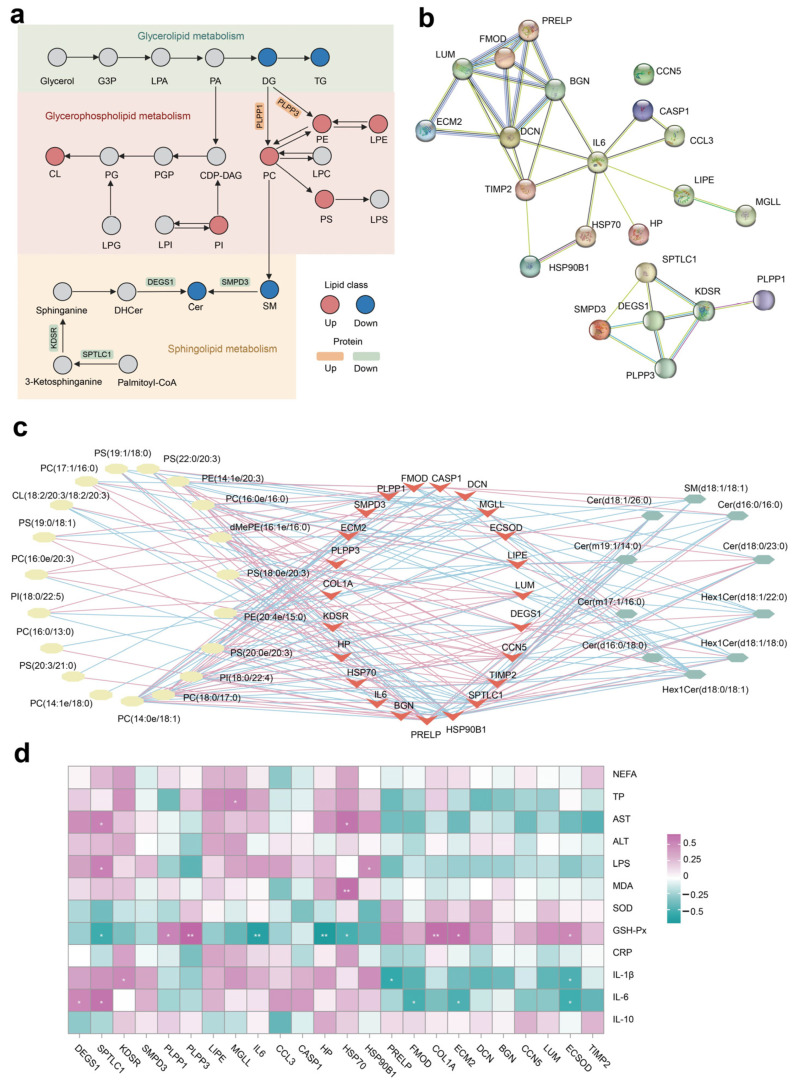
Integrated pathway and network analysis of the adipose tissue lipidome and proteome. (**a**) Diagram of lipid metabolism, including glycerolipid metabolism, glycerophospholipid metabolism, and sphingolipid metabolism; (**b**) protein–protein interaction (PPI) network of selected differentially expressed proteins (DEP); (**c**) Pearson’s correlation network between differential lipids (glycerophospholipid and sphingolipid species) and selected DEP; red lines represent positive, while blue lines indicate negative correlations (*p* < 0.05 and |r| > 0.50); (**d**) Pearson’s correlation analysis between selected DEP and serum biochemical parameters. * *p* < 0.05, ** *p* < 0.01. ALT, alanine aminotransferase; AST, aspartate aminotransferase; Cer, ceramide; CL, cardiolipin; CRP, C-reactive protein; dMePE, dimethylphosphatidylethanolamine; GSH-Px, glutathione peroxidase; IL, interleukin; LPS, lipopolysaccharide; MDA, malonaldehyde; NEFA, non-esterified fatty acid; PC, phosphatidylcholine; PE, phosphatidylethanolamine; PI, phosphatidylinositol; PS, phosphatidylserine; SM, sphingomyelin; SOD, superoxide dismutase; TP, total protein.

The integrated lipid metabolism pathway in adipose tissue showed that the upregulation of proteins PLPP1 and PLPP2 contribute to the increased SP abundance (e.g., PE, PC, and PS) (Figure 6a). The lower expression of protein SPTLC1, KDSR, DEGS1, and SMPD3 resulted in decreased Cer production in cows fed with NAR compared with CON cows. The PPI enrichment analysis demonstrates associations among selected DEP linked to ECM, inflammation, lipolysis, and heat stress (Figure 6b). Additionally, the PPI network indicates potential connections between DEPs associated with glycerophospholipid metabolism and sphingolipid metabolism. Nodes with higher degrees are considered more significant in the network. The network suggested that DCN, IL-6, and DEGS1 may be critical targets of NAR in adipose tissue. The differential SP and differential GP (top 20 of VIP) exhibited a high correlation between selected DEP (Figure 6c; *p* < 0.05 and |r| > 0.50). We also analyzed the association between serum metabolic status parameters and selected DEP (Figure 6d). The serum level of GSH-Px was positively correlated with ECSOD, ECM2, COL1A, PLPP1, and PLPP3 and was negatively correlated with HSP70, HP, IL6, and SPTLC1. Serum IL-6 was positively correlated with DEGS1 and SPTLC1 and was negatively correlated with FMOD, ECM2, and ECSOD.

## 4. Discussion

### 4.1. Milk Performance and Systemic Metabolic Status

Dietary supplementation with NAR had a significant effect on the milk performance of early-lactation cows. NAR supplementation led to higher yields of raw milk and protein, accompanied by lower SCC. These findings align with those of previous studies in which the supplementation with various plant flavonoids or polyphenols during the transition period increased milk production [8,9,33]. Despite a similar DMI between the two groups, it is likely that NAR supplementation improved energy utilization for milk production. However, no significant differences in prepartum or postpartum rumen volatile fatty acid (VFA) production were observed between the CON and NAR groups. The potential reduction of inflammation and metabolic stress may have contributed to the increased milk production. NAR supplementation may enhance milk protein yield by binding part of the dietary protein to polyphenols in the rumen, preventing microbial degradation and increasing protein flux in the small intestine. While naringin typically requires hydrolysis to its active form, naringenin, to exert health-promoting effects, previous studies have reported decreased milk SCC in cows fed citrus peel extract rich in naringin. Furthermore, naringenin administration via the intramammary route showed positive effects on milk SCC in mastitis-affected cows, suggesting improved mammary health status induced by naringenin.

Major physiologic, nutritional, metabolic, and immunologic changes occur within this time frame, as the production cycle of the cow shifts from a gestational nonlactating state to the onset of copious milk synthesis and secretion [34]. Inflammation plays a crucial role in these changes, mediated by various factors such as proinflammatory cytokines (e.g., TNF-α, IL-1β, IL-6, and INF-γ) [35]. Elevated levels of circulating IL-6 and IL-1β have been associated with transitional failure in dairy cows [36,37]. IL-10, an anti-inflammatory cytokine, acts to limit immune system-mediated damage and plays a role in host defense regulation [38]. Our findings indicate that NAR supplementation decreased serum IL-1β and IL-6 levels, while increasing IL-10 concentration, suggesting a potential reduction in systemic inflammation during the transition period. Transition cows often experience oxidative stress due to metabolic shifts [1], which has long been associated with compromised immune responses and increased health disorders around calving [39]. Supplementation with NAR led to a significant increase in serum GSH-Px levels and a decrease in MDA content, indicating a potential role for NAR in mitigating systemic oxidative stress.

As a consequence of metabolic stress, transition dairy cows usually develop an inflammation-like condition in the liver [40], which is evident from the induction of an acute phase response, characterized by the production of positive acute phase proteins such as serum SAA, Hp, or CRP, which compete with the production of essential liver proteins, known as negative acute phase proteins, including albumins, enzymes, and lipoproteins [41]. In our study, supplementation with NAR resulted in a significant decrease in serum CRP levels, while albumin remained unaffected by treatment. Additionally, levels of ALT and aspartate aminotransferase AST, markers for liver damage, were notably attenuated by NAR supplementation. These results suggest that feeding NAR may alleviate liver metabolic stress in periparturient cows.

### 4.2. Adipose Tissue Function and Lipid Metabolism

During the transition period, adipose tissue undergoes remodeling due to lipolysis, coinciding with a decline in insulin sensitivity in myocytes, hepatocytes, and adipocytes, redirecting energy towards milk production in the mammary gland [42]. As lactation progresses, bovine adipocytes become more responsive to insulin, resulting in reduced rates of lipolysis and increased lipogenesis [21]. While moderate insulin resistance in adipose tissue may support healthy and productive lactation during this period, excessive insulin resistance can predispose dairy cows to inflammation and metabolic dysfunction by limiting the adipose tissue’s ability to expand its energy-buffering capacity [42]. Additionally, lipolysis triggers a remodeling process within adipose tissue, which leads to drastically reduced adiponectin levels and increased biochemical blood indices associated with immune responses, such as cytokines, acute phase proteins, and heat shock protein after calving [43].

We observed that feeding with NAR significantly reduced serum NEFA concentration, suggesting inhibited adipose tissue lipolysis. Furthermore, the downregulation of adipose tissue proteins associated with lipolysis, such as LIPE and MGLL, observed in our study indicates a reduction in lipolysis in NAR-supplemented dairy cows. In line with our findings, cows supplemented with green tea and curcuma extract demonstrated reduced plasma NEFA during the first week after calving relative to that of the control cows [8]. Plasma NEFA was decreased 6 h after feeding in both early-lactation and mid-lactation cows fed citrus extract [18]. We observed that associations between NEFA and ceramide species, the central molecules of sphingolipid metabolism, play roles in the pathogenesis of obesity and metabolic diseases in non-ruminants [44]. The decrease in NEFA may be a primary factor leading to elevated adipose tissue concentrations and circulating ceramides, considering that NEFA is the primary substrate for ceramide biosynthesis.

Palmitic acid stands out as one of the most abundant fatty acids among circulating NEFA in cattle [45]. Ceramides, predominantly synthesized de novo from saturated fatty acids, particularly palmitate, originate in the endoplasmic reticulum. The synthetic process begins with the condensation of L-serine with palmitoyl-CoA, catalyzed by serine palmitoyltransferase, leading to the formation of 3-keto-dihydrosphingosine. Subsequently, 3-keto-dihydrosphingosine is reduced to dihydrosphingosine by 3-keto-dihydrosphingosine reductase [46]. The acylation of dihydrosphingosine by ceramide synthase isoforms generates dihydroceramide species [46]. Finally, dihydroceramide desaturase-1 desaturates the 4–5 position of the sphingolipid base backbone to produce various ceramide species [47]. Therefore, a reduced influx of palmitic acid could redirect into the de novo synthesis pathway, potentially resulting in lower concentrations of ceramide in both adipose tissue and blood. Our findings showed that NAR supplementation decreased protein levels of key enzymes of ceramide synthesis pathways in adipose tissue, including the de novo biosynthesis pathway (SPTLC1, KDSR, DEGS1) and sphingomyelin hydrolysis (SMPD3), resulting in a sphingolipidomic remodeling. Additionally, the results showed that the ceramide level of blood decreased synchronously with that of adipose tissue.

Sphingolipids, as bioactive lipids, possess the ability to modulate insulin sensitivity, cellular differentiation, and apoptosis in a tissue-specific manner [48]. Due to evolutionary conserved regulation, the underlying pathophysiological mechanisms of metabolic dysfunction, such as inflammatory signaling and inhibition of insulin signaling, accompanied by an increase in ceramide mediators, exhibit substantial overlap between humans and cattle [19,49,50,51,52]. Although systemic or adipose tissue insulin sensitivity was not quantified in our research, a significant decrease in inflammatory levels (e.g., concentration of IL-1β, IL-6, ASC, and NLRP3 and protein abundance of CCL3, CASP1, and HP) in adipose tissue was found in cows fed with NAR. A correlation analysis also suggested a possible interaction between sphingolipid metabolites and pro-inflammatory factors, both in the blood and the adipose tissue. Inflammatory cytokines such as IL-1β, IL-6, and TNF-α, released from hypertrophic adipocytes and M1 macrophages infiltrating adipose tissues, stimulate de novo ceramide synthesis from palmitate and the conversion of sphingomyelin into ceramide via sphingomyelinase activity [53]. Lipopolysaccharide, a highly immunogenic component of Gram-negative bacteria and a TLR4 activator, has been demonstrated to induce ceramide accumulation in serum [54]. Thus, the decrease in stimulatory factors (LPS and proinflammatory cytokines) could also contribute to the reduced ceramide accumulation in dairy cows fed with NAR. In nonruminants, a clear link exists between NLRP3 inflammasome and ceramide metabolism [55,56]. The NLRP3 inflammasome detects elevations in intracellular ceramide associated with lipotoxicity, leading to caspase-1 cleavage in macrophages and adipose tissue [57]. Our findings suggest that enhancing sphingolipid homeostasis could be crucial in reducing systemic and adipose tissue inflammation levels by inhibiting the NLRP3 pathway.

Furthermore, we observed that feeding with NAR increased the adipose tissue level of T-AOC and tended to enhance SOD, indicating that naringin may alleviate oxidative stress in adipose tissue. On the one hand, even though ceramides are essential for the normal function of mitochondria, excessive levels of mitochondrial ceramides can lead to mitochondrial dysfunction and impair respiratory capacity by generating reactive oxygen species (ROS) and causing increased oxidative stress, reducing ATP, disrupting the electron transport chain, causing apoptosis, and altering the permeability of the mitochondrial outer membrane [58]. On the other hand, H_2_O_2_, an inflammatory oxidant, and hypoxia, which causes irreversible impairment in multiple cellular systems, have been used to indicate that oxidant-induced apoptosis is mediated by the SM-ceramide pathway [59]. Indeed, if ROS can stimulate ceramide production, ceramide may inhibit isolated mitochondrial electron transport at complex III, increasing reactive oxygen species [60]. Interestingly, we observed reductions in the abundances of two heat shock proteins (HSP70 and HSP90B1) in the adipose tissue of dairy cows fed with NAR. This experiment was conducted in Beijing from August to September 2022, during periods of high temperatures. Although we did not record the temperature and humidity of the barns at that time, it is highly likely that the cows experienced heat stress. Oxidative stress is a well-documented consequence of heat stress, resulting in damage at both the cellular and mitochondrial levels [61]. During heat stress, one of the most commonly observed heat shock proteins to increase is HSP70 [61]. Hence, the beneficial impact of NAR on ceramide metabolism and heat stress serves to mitigate the adipose tissue oxidative stress of transition cows.

Adiponectin, primarily secreted by adipose tissue and circulating at high concentrations in the blood, plays a crucial role in regulating energy metabolism by enhancing insulin sensitivity, promoting adipogenesis, and facilitating lipid storage in adipose tissue. Additionally, it stimulates lipogenesis in the hepatocytes and enhances fatty acid β-oxidation in the myocytes and hepatocytes in humans [62]. In our study, although serum adiponectin levels did not differ between the two groups, the adiponectin concentration in adipose tissue was lower in cows fed with NAR compared to those fed with CON. The upregulation of adiponectin in the adipose tissue of periparturient dairy cows may imply improved insulin sensitivity, leading to enhanced glucose uptake, reduced lipolysis [63], and the attenuated inflammatory response of monocytes [64]. Adiponectin stimulates ceramidase activity linked to its receptors, AdipoR1 and AdipoR2 [65], promoting ceramide catabolism and increasing the formation of sphingosine-1-phosphate, an antiapoptotic metabolite [65]. Furthermore, our study revealed a negative correlation between adiponectin concentrations and ceramide levels, providing further evidence for the existence of an adiponectin–ceramide axis in the adipose tissue of dairy cows. Therefore, feeding with NAR increased adiponectin secretion, potentially reducing the accumulation of ceramides, and regulating downstream events such as inflammation, oxidative stress, and insulin resistance.

We found a significantly increased level of glycerophospholipids (e.g., PS, PC, PE, PI) in the adipose tissue of cows fed with NAR. Upregulating two essential proteins (PLPP1 and PLPP3) in the glycerophospholipid metabolism pathway may promote the synthesis of glycerophospholipids. Glycerophospholipids are critical components of cell membranes, and their upregulation may enhance membrane stability and facilitate cellular processes such as lipid transport, signaling, and receptor function [66]. The observed increase in PS, PI, PE, and PC levels may reflect membrane dynamics or lipid metabolism adaptations within adipose tissue [67]. Glycerophospholipids are also involved in the signaling pathways that regulate immune responses [68]. Increased PS, PI, PE, and PC levels may modulate immune signaling pathways within adipose tissue, promoting a balanced immune response and reducing inflammation.

Additionally, glycerophospholipids are antioxidants that protect cells from oxidative damage [69]. The upregulation of glycerophospholipids may enhance the antioxidant defense mechanisms within adipose tissue, reducing oxidative stress and supporting immune cell function. However, unlike the synchronized downregulation observed in serum sphingolipids and adipose tissue sphingolipids, serum glycerophospholipids did not exhibit synchronous upregulation with adipose tissue. This observation underscores the need for further investigation.

Surprisingly, this study found that feeding NAR resulted in a significant upregulation of ECM-related proteins in adipose tissue, and multiple differential ECM proteins were found to interact with IL-6. Lipolysis represents a catabolic process that significantly reduces adipose tissue mass in transition cattle [70]. Less is known about the expression of ECM proteins and their effect on adipose tissue metabolic function in transition dairy cows [71]. The ECM provides structural support and regulates cellular processes such as adipogenesis, lipolysis, and adipokine secretion. We assumed that the upregulation of ECM-related proteins suggests an active remodeling process within the adipose tissue, induced by NAR. This remodeling could involve structural modifications or alterations in the composition of the ECM, potentially leading to improved tissue integrity and function.

Overall, altered immunological activity in adipose tissue may contribute to energetic savings, resulting in the enhanced milk yield observed in this study. An immune response increases energy demand by up to 55% [72], or the equivalent of 1 kg of glucose utilized in a 12 h period [73], so alterations in immune response may conserve energy, without affecting DMI. In this study, the altered immune response may have resulted in energetic savings that improved milk production without affecting DMI, although this was not directly measured. Further research could lead to better quantification of the benefits of immunomodulation of cows during the periparturient period. Future studies should also focus on measuring and quantifying the energetic use of transition dairy cows fed with NAR.

## 5. Conclusions

NAR supplementation in transition cows improved liver function, reduced systemic inflammation, enhanced antioxidative capacity, and improved early-lactation performance. NAR also increased adipose tissue adiponectin production, inhibited excessive lipolysis, and reduced adipose tissue inflammation, heat stress, and oxidative stress, indicating an attenuation of metabolic disorders. Lipidomic and proteomic data suggest that NAR directly impacts lipid metabolism pathways, decreasing sphingolipids and increasing glycerophospholipids in adipose tissue. These lipid changes likely contribute to reduced inflammation and oxidative stress. NAR supplementation also lowered lipolysis and key enzymes involved in ceramide synthesis, while reducing stimulatory factors like LPS and IL-6, resulting in a favorable lipid profile. The reduction in serum and adipose tissue sphingolipids highlights their role in mediating the metabolic benefits of NAR.

## Figures and Tables

**Table 1:** Effects of dietary supplementation with naringin on feed intake and lactation performance.

Item ^1^	Treatment ^2^		SEM	*p*-Value ^3^		
	CON	NAR		Trt	Time	Trt × Time
Prepartum DMI, kg/d	12.9	12.6	0.25	0.542	<0.001	0.509
Postpartum DMI, kg/d	20.3	20.2	0.14	0.646	<0.001	0.873
Milk yield, kg/d	42.0	45.7	1.17	0.038	<0.001	0.978
3.5% FCM ^4^, kg/d	45.1	48.6	1.44	0.106	0.012	0.872
ECM ^5^, kg/d	45.5	48.3	1.60	0.242	0.003	0.689
Milk composition						
Fat, %	4.12	3.91	0.194	0.384	0.001	0.371
Fat, kg/d	1.69	1.75	0.084	0.564	0.105	0.594
Protein, %	3.23	3.36	0.051	0.086	<0.001	0.083
Protein, kg/d	1.32	1.51	0.041	0.002	0.347	0.887
Lactose, %	5.09	5.14	0.046	0.461	0.005	0.031
Lactose, kg/d	2.14	2.35	0.073	0.061	<0.001	0.997
MUN, mg/dL	13.5	13.9	0.503	0.560	<0.001	0.478
SCC, ×103 cells/mL	197	130	22.7	0.018	0.003	0.929
Feed efficiency						
Milk yield/DMI	1.98	2.17	0.059	0.028	0.910	0.983
3.5% FCM/DMI	2.15	2.32	0.086	0.169	0.053	0.495
ECM/DMI	2.14	2.34	0.078	0.079	0.005	0.662

^1^ DMI, dry matter intake; FCM, fat corrected milk; ECM, energy corrected milk; MUN, milk urea nitrogen; SCC, somatic cell count. ^2^ CON, no supplemental naringin (n = 18); NAR = 30 g/d per cow of naringin. ^3^ Trt = treatment. ^4^ ECM was calculated as: ECM = [(0.327 × milk yield) + (12.95 × fat yield) + (7.2 × protein yield)]. ^5^ 3.5% FCM was calculated as: 3.5% FCM = [(0.432 × milk yield) + (16.23 × fat yield)].

**Table 2:** Effects of dietary supplementation with naringin on serum glucose and lipid metabolism biomarkers.

Item ^1^	Treatment ^2^		SEM	*p*-Value ^3^		
	CON	NAR		Trt	Time	Trt × Time
NEFA, mmol/L	0.674	0.547	0.0483	0.028	<0.001	0.462
BHBA, mmol/L	0.586	0.567	0.0264	0.613	<0.001	0.182
Glucose, mmol/L	3.37	3.46	0.077	0.386	0.003	0.778
Insulin, uU/mL	13.6	13.4	0.14	0.228	<0.001	0.362
TG, mmol/L	0.0911	0.0913	0.00512	0.980	<0.001	0.973
TC, mmol/L	2.10	2.03	0.161	0.753	<0.001	0.653
LDL-C, mmol/L	0.712	0.597	0.0591	0.173	<0.001	0.946
Leptin, μg/L	4.07	4.22	0.084	0.222	0.225	0.848
Adiponectin, mg/L	53.8	57.5	2.450	0.296	0.037	0.872

^1^ NEFA, non-esterified fatty acid; BHBA, β-hydroxybutyric acid; TG, triglyceride; TC, total cholesterol; LDL-C, low-density lipoprotein cholesterol. ^2^ CON, no supplemental naringin (n = 18); NAR = 30 g/d naringin. Serum samples were collected on −7 d relative to calving and on 2, 14, or 28 d postpartum. ^3^ Trt = treatment.

**Table 3:** Effects of dietary supplementation with naringin on liver function indices.

Item ^1^	Treatment ^2^		SEM	*p*-Value ^3^		
	CON	NAR		Trt	Time	Trt × Time
TP, g/L	67.5	64.4	1.06	0.056	<0.001	0.019
ALB, g/L	36.3	36.6	0.60	0.722	0.266	0.413
AST, U/L	77.5	63.1	3.72	0.012	0.007	0.008
ALT, U/L	28.6	21.7	1.47	0.002	<0.001	0.011

^1^ TP, total protein; ALB, albumin; AST, aspartate aminotransferase; ALT, alanine aminotransferase. ^2^ CON, no supplemental naringin (n = 18); NAR = 30 g/d per cow of naringin. Serum samples were collected on −7 d relative to calving and on 2, 14, or 28 d postpartum. ^3^ Trt = treatment.

**Table 4:** Effects of dietary supplementation with naringin on serum endotoxin, antioxidant status, and proinflammatory factors.

Item ^1^	Treatment ^2^		SEM	*p*-Value ^3^		
	CON	NAR		Trt	Time	Trt × Time
LPS, EU/mL	0.351	0.294	0.0077	<0.001	<0.001	<0.001
MDA, umol/L	4.12	3.31	0.203	0.010	<0.001	0.406
SOD, U/mL	74.5	76.8	0.86	0.062	<0.001	0.248
GSH-Px, U/mL	370	383	4.0	0.029	<0.001	0.092
CRP, mg/mL	12.7	12.0	0.20	0.019	<0.001	0.512
IL-1β, ng/L	78.5	69.7	1.79	0.004	0.737	0.824
IL-2, ng/L	262	250	7.2	0.242	0.142	0.985
IL-6, ng/L	15.8	12.6	0.45	<0.001	0.019	0.636
IL-10, pg/mL	11.1	11.9	0.22	0.012	<0.001	0.329
TNF-α, ng/L	256	246	5.2	0.190	0.507	0.884

^1^ LPS, lipopolysaccharide; MDA, malonaldehyde; SOD, superoxide dismutase; GSH-Px, glutathione peroxidase; CRP, C-reactive protein; IL, interleukin; TNF-α, tumor necrosis factor α. ^2^ CON, no supplemental naringin (n = 18); NAR = 30 g/d per cow of naringin. Serum samples were collected on −7 d relative to calving and on 2, 14, or 28 d postpartum. ^3^ Trt = treatment.

**Table 5:** Effects of dietary supplementation with naringin on biochemical parameters in adipose tissue.

Item ^1^	Treatment ^2^		SEM	*p*-Value ^3^		
	CON	NAR		Trt	Time	Trt × Time
Adiponectin, mg/g	0.930	1.036	0.0190	0.001	0.003	0.732
IL-1β, pg/mg	0.755	0.680	0.0131	<0.001	<0.001	0.147
IL-6, pg/mg	4.17	4.00	0.039	0.012	<0.001	0.598
TNF-α, pg/mg	2.33	2.21	0.028	0.138	<0.001	0.153
SOD, U/mg	2.46	2.55	4.0	0.064	<0.001	0.907
T-AOC, U/mg	0.417	0.474	0.0066	<0.001	<0.001	0.037
ASC, pg/mg	0.563	0.492	0.0135	0.002	<0.001	0.470
NLRP3, pg/mg	5.88	5.66	0.093	0.050	<0.001	0.723

^1^ IL, interleukin; TNF-α, tumor necrosis factor α; SOD, superoxide dismutase; T-AOC: total antioxidant capacity; ASC, apoptosis-associated speck like protein containing a CARD; NLRP3, NOD-like receptor thermal protein domain-associated protein 3. ^2^ CON, no supplemental naringin (n = 18); NAR = 30 g/d per cow of naringin. Subcutaneous adipose tissue samples were collected on 2, 14, 28 d postpartum. ^3^ Trt = treatment.

## Data Availability

The raw data supporting the conclusions of this article will be made available by the authors on request.

## Supplementary Materials

The following supporting information can be downloaded at: https://www.mdpi.com/article/10.3390/antiox13060638/s1, Table S1: Experimental diets (% of DM unless otherwise indicated) fed to cows during the dry and lactation period. Table S2: Differential lipid species in the adipose tissue samples between CON and NAR cows. Table S3: Differential lipid species in the adipose tissue samples between CON and NAR cows. Table S4: Differentially expressed proteins in adipose tissue of dairy cows. Figure S1: Relative lipid class composition of (a) serum and (b) adipose tissue samples. Figure S2: Lipid class variations observed in (a) serum and (b) adipose tissue samples.
