# Supplementary material for: Dietary Supplementation with Naringin Improves Systemic Metabolic Status and Alleviates Oxidative Stress in Transition Cows via Modulating Adipose Tissue Function: A Lipid Perspective
Source: Antioxidants (Basel). 2024 May 24;13(6):638. doi: 10.3390/antiox13060638 (PMC11200899; doi:10.3390/antiox13060638)
Supplement: Supplementary file 1 [file antioxidants-13-00638-s001.zip › antioxidants-3009960-supplementary/antioxidants-3009960-supplementary-for publish/antioxidants-3009960-supplementary-file S1.pdf]

antioxidants

MDPI

Table S1. Experimental diets (% of DM unless otherwise indicated) fed to cows during the dry and lactation period.

Item [1]	Prepartum	Early lactation
Ingredient, % of DM		
Corn silage	25.7	30.4
Alfalfa silage	-	7.84
Oat Hay	45.6	-
Alfalfa hay	-	10.4
Soybean meal	7.68	16.2
Soybean hull	7.74	3.65
Crushed corn	3.88	9.46
Corn steam flakes	-	9.30
Whole cottonseed	-	5.36
Corn gluten meal	4.08	-
Sugarcane molasses	-	2.59
DCAD supplement	1.01	1.01
Mineral and vitamin mix	4.31[2]	3.70[3]
Chemical composition, % of DM		
DM, as fed %	51.2	47.8
CP	15.6	17.5
NFC[4]	33.2	38.5
NDF	43.5	33.2
ADF	23.5	19.3
EE	3.48	5.10
Ash	5.10	5.68
NE_L (Mcal/kg of DM)	1.38	1.69
DCAD[5]	−98.1	291

[1] DM = dry matter; DCAD = dietary cation-anion difference; CP = crude protein; NFC = non-fiber carbohydrates; NDF = neutral detergent fiber; ADF = acid detergent fiber; EE = ether extract; NE_L = net energy for lactation. [2] The mineral mix contained (per kg): 44 g of calcium carbonate, 19 g of sodium chloride, 16 g of calcium sodium phosphate, 12 g of magnesium oxide, 3 g of magnesium sulfate, 400,000 IU of vitamin A, 65,000 IU of vitamin D3, 4,000 mg of vitamin E, 250 mg of vitamin C, 80 mg of vitamin B1, 40 mg of vitamin B2, 20 mg of vitamin B6, 200 μg of vitamin of B12, 5,000 mg of niacinamide, 80 mg of calcium d-pantothenate, 16,600 μg of biotin, 16,500 mg of choline chloride, 5,000 mg of zinc [4,500 mg as solid glycine zinc chelate hydrate (solid) and 500 mg as zinc oxide], 5,000 mg of manganese [1,500 mg as glycine-manganese chelate hydrate and 3,500 mg as manganese(II)-oxide], 1,000 mg of copper [750 mg as copper(II) glycine chelate hydrate (solid) and 250 mg as copper(II) sulfate pentahydrate], 150 mg of iodine as calcium iodate (anhydrous), 15 mg of cobalt as coated cobalt(II) carbonate granules, 25 mg of selenium [3 mg as selenium yeast from Saccharomyces cerevisiae NCYC R397 (inactivated) and 22 mg as sodium selenite]. [3] The mineral mix contained (per kg): 9,000 IU of vitamin A, 1,000 IU of vitamin D3, 10 mg of vitamin E, 13 mg of copper-(II)-sulfate, pentahydrate, 0.75 mg of iodine as calcium iodate (anhydrous), 0.15 mg of cobalt as coated cobalt(II) carbonate granules, 30 mg of manganese as manganese(II)-oxide, 45 mg of zinc as zinc oxide, and 0.12 mg of selenium as sodium selenite. [4] NFC = DM − (ash + CP + EE + NDF). [5] DCAD (mEq/kg of DM) = (Na + K) − (Cl + S), where the unit of Na, K, Cl, and S is mEq/kg of DM.

Table S2. Differential lipid species in serum samples between CON and NAR cows.

Lipid Species	VIP	*p*-Value	FC	Regulate
Hex2Cer(d16:0/18:1)	1.6578	0.0311	1.0187	up
PS(20:0/18:1)	1.5125	0.0075	1.0127	up
MGDG(18:0e/15:0)	1.4683	0.0428	1.0174	up
MePC(8:1e/21:0)	3.2984	0.0282	0.8928	down
PC(12:0e/20:4)	3.1806	0.0249	0.8976	down
PE(16:0p/22:5)	3.0642	0.0237	0.8946	down
DG(20:0/16:0)	2.8737	0.0078	0.8921	down
LPC(12:0)	2.6787	0.0072	0.9043	down
PI(18:1/18:1)	2.6256	0.0289	0.9518	down
TG(14:1e/16:0/16:0)	2.4813	0.0244	0.9332	down
TG(16:2e/16:0/17:0)	2.4217	0.0042	0.9482	down
PI(18:0/18:0)	2.3542	0.0066	0.9527	down
DG(18:2e/18:0)	2.2465	0.0105	0.9567	down
TG(16:1/16:1/18:2)	2.2465	0.0247	0.9536	down
ZyE(17:1)	2.2417	0.0362	0.9415	down
TG(16:2e/16:0/16:0)	2.2153	0.0242	0.9527	down
PC(16:1e/15:0)	2.2032	0.0075	0.9753	down
PS(18:0e/16:0)	2.2032	0.0075	0.9753	down
Cer(d18:0/23:0)	2.1880	0.0098	0.9482	down
PI(16:0/16:0)	2.1817	0.0213	0.9669	down
Cer(d18:0/22:0)	2.1663	0.0242	0.9678	down
Cer(d18:0/24:0)	1.9335	0.0316	0.9469	down
MePC(14:0e/16:0)	1.9267	0.0194	0.9761	down
SM(d14:0/16:0)	1.9215	0.0257	0.9622	down
PE(16:0p/18:1)	1.9162	0.0257	0.9633	down
MePC(18:0/18:2)	1.7629	0.0353	0.9606	down
SM(t18:1/23:6)	1.6779	0.0305	0.9711	down
BisMePA(25:1/6:0)	1.6400	0.0442	0.9637	down
PC(6:0/22:0)	1.6179	0.0481	0.9641	down
Cer(d18:1/24:0)	1.5564	0.0157	0.9845	down
MePC(14:1e/18:2)	1.5564	0.0330	0.9812	down
Cer(d18:1/23:0)	1.5394	0.0443	0.9832	down
SiE(18:2)	1.5379	0.0290	0.9721	down
Co(Q10)	1.5129	0.0358	0.9728	down
MePC(20:3e/11:4)	1.4914	0.0474	0.9805	down
SM(t18:1/18:1)	1.4687	0.0273	0.9808	down
SM(t18:1/22:5)	1.4581	0.0276	0.9821	down
MePC(18:3e/17:0)	1.4418	0.0341	0.9841	down
SM(d18:1/21:0)	1.3528	0.0333	0.9887	down
PC(8:1e/23:0)	1.0885	0.0014	0.9835	down
PC(14:0e/16:0)	1.0520	0.0292	0.9848	down
PC(16:1/16:1)	1.0318	0.0437	0.9853	down
LPC(20:5)	1.0155	0.0241	0.9845	down

VIP: variable importance in the projection; FC: fold change; CON: cows without naringin; NAR: cows fed with naringin.

Table S3. Differential lipid species in the adipose tissue samples between CON and NAR cows.

Lipid Species	VIP	*p*-Value	FC	Regulate
TG(15:0/6:0/16:1)	3.2621	0.0285	1.0956	up
PS(19:0/18:1)	2.5013	0.0100	1.0726	up
PC(18:0/17:0)	2.1402	0.0002	1.034	up
PS(22:0/20:3)	2.0757	0.0157	1.0432	up
Cer(m19:1/14:0)	2.0704	0.0109	1.0343	up
CL(18:2/20:3/18:2/20:3)	1.976	0.0083	1.047	up
TG(18:4/14:4/19:0)	1.8935	0.0219	1.0267	up
TG(10:0/14:1/14:1)	1.8842	0.0174	1.0253	up
PI(18:0/22:4)	1.8818	0.0043	1.0305	up
PS(20:0e/20:3)	1.8724	0.0080	1.0328	up
PC(16:0/13:0)	1.8610	0.0181	1.033	up
PS(18:0e/20:3)	1.8583	0.0142	1.0345	up
DG(18:2e/18:3)	1.8375	0.0360	1.0259	up
PE(20:4e/15:0)	1.8252	0.0157	1.0332	up
PC(14:1e/18:0)	1.8125	0.0167	1.0274	up
PC(16:0e/20:3)	1.8053	0.0122	1.0315	up
DG(16:2e/14:1)	1.8013	0.0269	1.0255	up
PS(19:1/18:0)	1.7321	0.0051	1.0275	up
PC(17:1/16:0)	1.6906	0.0175	1.03	up
PS(20:3/21:0)	1.6642	0.0177	1.0301	up
PC(18:0e/20:3)	1.6621	0.0331	1.0341	up
PE(14:1e/20:3)	1.6556	0.0217	1.0273	up
PI(18:0/22:5)	1.6357	0.0189	1.0276	up
PE(16:1/18:1)	1.6349	0.0236	1.0272	up
PE(20:0/20:4)	1.5930	0.0023	1.0215	up
PE(16:0/16:1)	1.5757	0.0036	1.0223	up
PC(20:4e/17:0)	1.5448	0.0439	1.0228	up
PE(17:0/20:4)	1.5399	0.0198	1.0238	up
PS(20:3/22:5)	1.5242	0.0365	1.0275	up
PE(16:1/20:4)	1.5146	0.0281	1.0232	up
PC(16:0/19:0)	1.5082	0.0186	1.0187	up
PS(22:4/21:0)	1.5067	0.0303	1.0284	up
PS(20:2/22:5)	1.4888	0.0049	1.0189	up
PE(17:0/18:1)	1.4527	0.0046	1.0163	up
PE(17:1/16:0)	1.4489	0.0048	1.0202	up
PC(18:1e/18:2)	1.4389	0.0327	1.0181	up
PC(16:1/19:0)	1.4370	0.0367	1.0168	up
PS(18:0/22:5)	1.4144	0.0046	1.0165	up
PE(18:0/22:5)	1.4126	0.0305	1.0193	up
dMePE(16:0/22:5)	1.4126	0.0305	1.0193	up
Cer(d18:1/26:0)	1.3890	0.0221	1.0205	up
PE(16:0/22:4)	1.3816	0.0066	1.0168	up
PS(20:0/20:3)	1.3625	0.0231	1.0197	up
PS(20:2/20:3)	1.3598	0.0493	1.0179	up
CL(18:2/18:1/20:3/18:2)	1.3369	0.0428	1.0241	up
PC(16:1/13:0)	1.3271	0.0397	1.0172	up
PS(18:1/20:3)	1.3181	0.0389	1.0193	up
PS(20:3/22:4)	1.2927	0.0079	1.0169	up
PC(17:0/18:1)	1.2654	0.0104	1.0133	up
PE(18:0/20:3)	1.2449	0.0325	1.0124	up
CL(24:0/16:0/18:0/18:0)	1.2413	0.0208	1.0159	up
TG(18:0/15:0/16:0)	1.2154	0.0382	1.0138	up
PC(19:1/18:0)	1.2141	0.0214	1.0167	up
PS(18:0/22:4)	1.199	0.0154	1.0145	up

PI(16:0/18:1)	1.1586	0.0467	1.0153	up
PI(16:0/20:4)	1.1189	0.0401	1.0138	up
PI(18:0/20:4)	1.0692	0.0183	1.0103	up
dMePE(17:0/18:1)	1.0668	0.0215	1.0118	up
PIP2(18:2e/19:0)	1.0579	0.0199	1.0113	up
TG(16:0/14:0/17:1)	4.4852	0.0229	0.8374	down
TG(16:1/6:0/14:1)	4.0538	0.0071	0.8964	down
TG(11:0/16:0/18:1)	3.940	0.0055	0.8991	down
OAHFA(20:4/18:0)	3.5222	0.0188	0.8683	down
OAHFA(16:0/19:0)	3.4535	0.0204	0.8720	down
OAHFA(16:0/16:0)	3.1206	0.0363	0.8927	down
OAHFA(16:0/18:0)	3.0970	0.0329	0.8968	down
OAHFA(18:1/19:0)	3.0937	0.0466	0.8893	down
Hex1Cer(d18:1/18:0)	2.4295	0.0060	0.9545	down
Hex1Cer(d18:0/18:1)	2.3772	0.0031	0.9532	down
Hex1Cer(d18:1/22:0)	2.1486	0.0095	0.9656	down
TG(18:1/18:1/22:4)	2.0577	0.0010	0.9738	down
PC(14:0e/18:1)	2.0449	0.0023	0.9750	down
DG(18:0/20:3)	2.0302	0.0062	0.9733	down
dMePE(16:1e/16:0)	1.8360	0.0122	0.9659	down
TG(16:0/14:4/18:1)	1.7729	0.0062	0.9799	down
TG(18:0/18:1/22:4)	1.7124	0.0033	0.9806	down
PC(16:0e/16:0)	1.7061	0.0122	0.9778	down
Cer(d18:0/23:0)	1.7048	0.0041	0.9729	down
TG(18:1/18:2/20:3)	1.6731	0.0010	0.9844	down
Cer(m17:1/16:0)	1.639	0.0290	0.9756	down
TG(18:1/18:1/20:3)	1.6219	0.0004	0.9863	down
Cer(m18:0/16:0)	1.6209	0.0196	0.9802	down
Cer(d16:0/16:0)	1.5978	0.0008	0.9865	down
TG(12:0e/6:0/20:3)	1.5940	0.0219	0.9802	down
TG(12:0e/6:0/22:6)	1.5940	0.0219	0.9802	down
PC(16:1e/16:0)	1.5458	0.0178	0.9783	down
TG(18:1/18:2/20:4)	1.4827	0.0430	0.9788	down
PE(16:1e/16:0)	1.4243	0.0425	0.9762	down
SM(d18:1/18:1)	1.3814	0.0051	0.9875	down
DG(18:0/20:4)	1.3596	0.0162	0.9863	down
PE(16:0p/22:4)	1.3528	0.0396	0.9845	down
BisMePA(16:2e/22:4)	1.3528	0.0396	0.9845	down
MePC(14:1e/20:4)	1.2657	0.0279	0.9861	down
TG(16:1/14:0/14:1)	1.2196	0.0161	0.9890	down
TG(16:1/12:0/14:0)	1.1934	0.0372	0.9892	down
Cer(d16:0/18:0)	1.1101	0.0430	0.9899	down
PE(16:1e/22:4)	1.1062	0.0487	0.9904	down
dMePE(16:1/18:1)	1.0996	0.0090	0.9897	down
DG(10:0e/16:0)	1.0841	0.0331	0.9912	down
TG(20:0/18:1/18:1)	1.0501	0.0245	0.9922	down

VIP: variable importance in the projection; FC: fold change; CON: cows without naringin; NAR: cows fed with naringin.

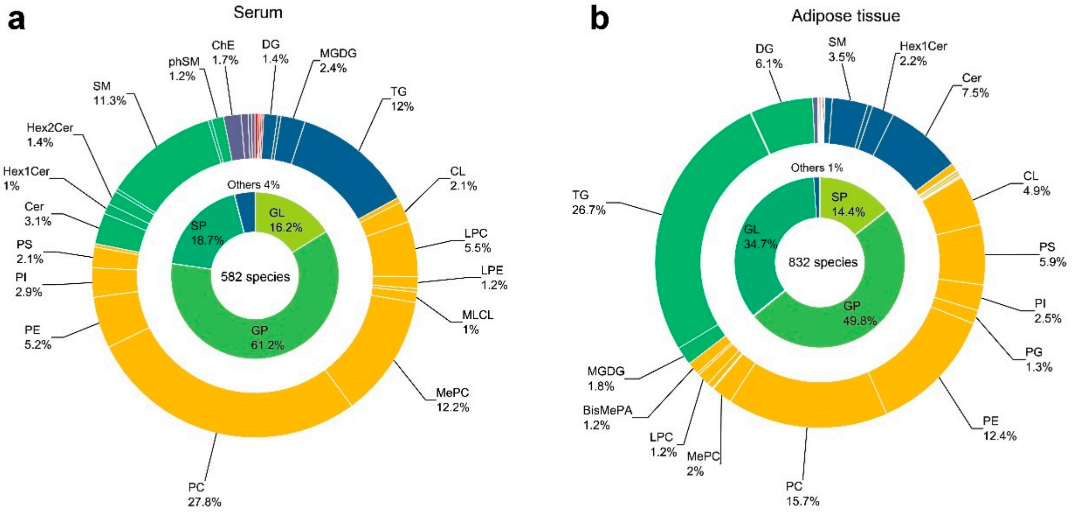

Figure S1. Relative lipid class composition of (a) serum and (b) adipose tissue samples. GL, glycerolipid; DG, diradylglycerols; MGDG, monogalactosyldiacylglycerol; TG, Triacylglycerol; GP, glycerophospholipid; CL, cardiolipin; LPC, lysophosphatidylcholine; LPE, lysophosphatidylethanolamine; MLCL, monolysocardiolipin; MePC, methylphosphatidylcholine; PC, phosphatidylcholine; PE, phosphatidylethanolamine; PI, phosphatidylinositol; PS, phosphatidylserine; SP, sphingolipid; Cer, ceramide; Hex1Cer, hexosylceramide; Hex2Cer, dihexosylceramide; SM, sphingomyelin; phSM, phosphorylated sphingomyelin; ChE, cholesteryl ester.

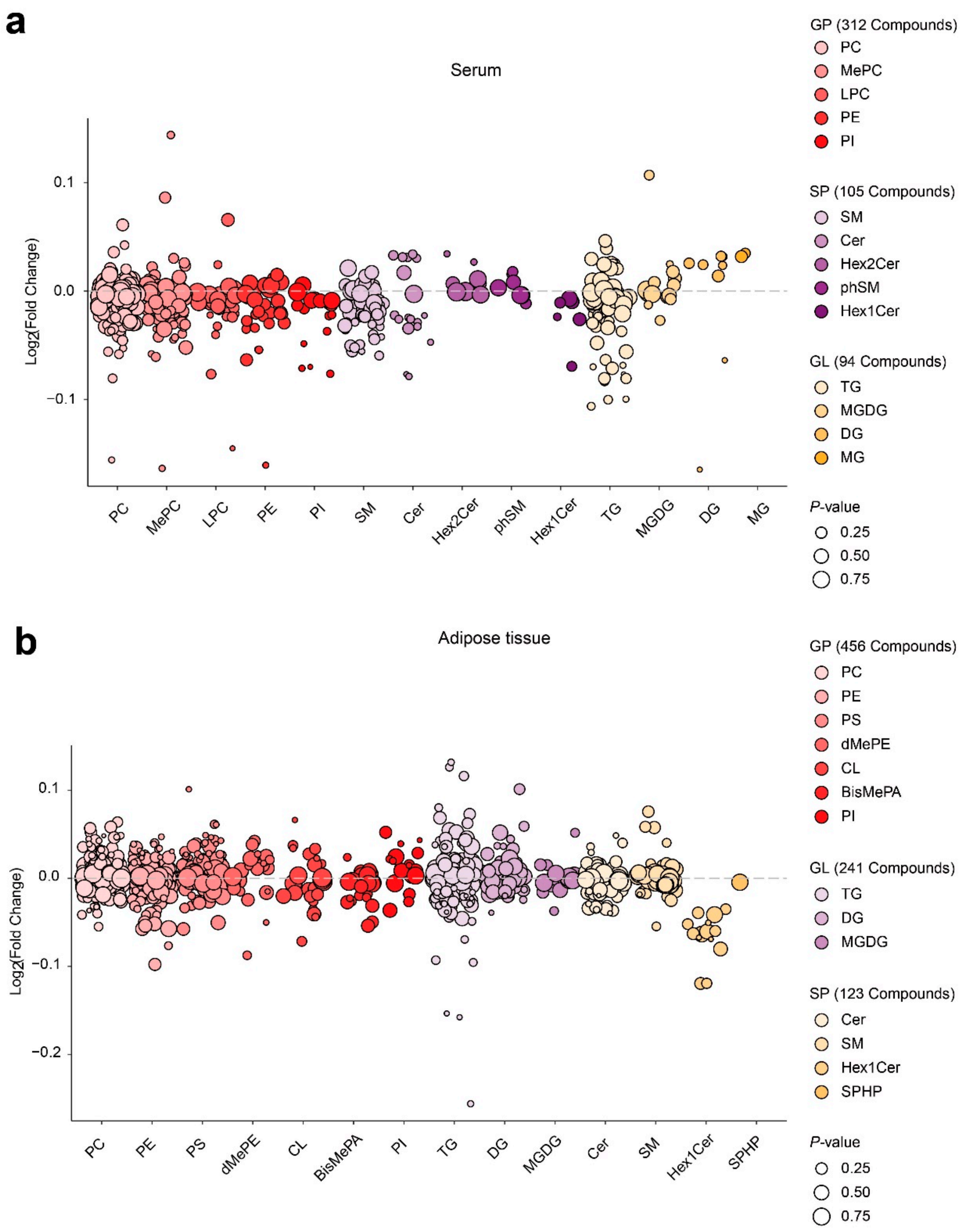

Figure S2. Lipid class variations observed in (a) serum and (b) adipose tissue samples. BisMePA, bismethyl phosphatidic acid; Cer, ceramide; CL, cardiolipin; DG, diradylglycerols; dMePE, dimethylphosphatidylethanolamine; GL, glycerolipid; GP, glycerophospholipid; Hex1Cer, hexosylceramide; Hex2Cer, dihexosylceramide; LPC, lysophosphatidylcholine; MePC, methylphosphatidylcholine; MG, monoacylglycerol; MGDG, monogalactosyldiacylglycerol; PC, phosphatidylcholine; PE, phosphatidylethanolamine; PI, phosphatidylinositol; PS, phosphatidylserine; SM, sphingomyelin; SP, sphingolipid; SPHP, Sphingophosphonolipid; TG, Triacylglycerol.
